# A Modified Technique of Fixation for Proximal Femoral Valgus Osteotomy in Abnormal Bone: A Report of Two Cases

**DOI:** 10.5704/MOJ.1707.014

**Published:** 2017-07

**Authors:** S Logheswaren, AR Sulaiman, I Munajat

**Affiliations:** Department of Orthopaedics, Universiti Sains Malaysia, Kubang Kerian, Malaysia

**Keywords:** valgus osteotomy, modified technique, abnormal bone

## Abstract

The ideal size of intramedullary device to fix corrective osteotomy of proximal femur in abnormal bone in children and small patients may not be easily available. We report the successful use of Rush rod in combination with multiple Kirschner wires to fix the corrective osteotomy of coxa vara and shepherd crook deformity in two patients with osteogenesis imperfecta and fibrous dysplasia. The union was achieved on time, neck shaft angle and rotation were maintained.

## Introduction

Coxa vara and shepherd’s crook deformity may occur following multiple stress fractures or mal-union. It is especially common in abnormal bone like fibrous dysplasia and osteogenesis imperfecta. Correction of these deformities will improve limb length discrepancy. It will also improve the hip abductor mechanism by correcting the working length of abductor muscle thus correcting Trendelenburg gait. Fixation for corrective osteotomy for coxa vara or shepherd’s crook deformity in abnormal bone like osteogenesis imperfecta and fibrous dysplasia in children and small patients is challenging due to difficulties to obtain appropriate size of intramedullary nail to fit a small bone. Plates and screws are not the preferred method due to stress points on both ends that may lead to fractures. We report the successful modification of the technique described by Fassier *et al*^1^ by Rush rod in combination with Kirschner wires to fix the corrective osteotomy of coxa vara in osteogenesis imperfecta and fibrous dysplasia patients.

## Case Report

**Fig. 1:**
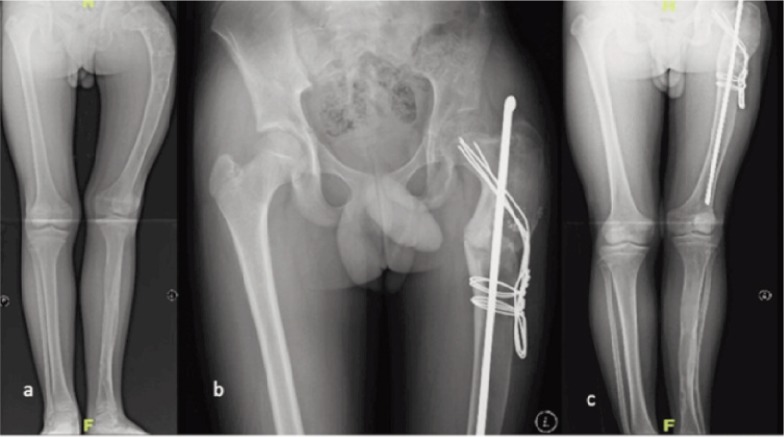
Series of radiographs of the first patient, the 14-year old boy with fibrous dysplasia: (a) Shepherd’s crook deformity of left femur with loss of the normal trabecular pattern and ground-glass appearance (b) Immediate post-operative radiograph shows valgus osteotomy and intramedullary Rush rod and Kirschner wires in the neck bent and held to side of femoral shaft distal to osteotomy site (c) The radiograph at 22 months after surgery shows the preservation of neck shaft angle with fixation devices in place.

The first patient was a 14-year old boy with fibrous dysplasia who presented with limping due to worsening deformity of the left femur (Fig. 1). The Trendelenburg test was positive. The left femur was shorter by 2.5cm. Surgical correction was achieved using the modified surgical method as described below. A Rush rod size 1F (6.5mm) and four Kirschner wires size 1.4mm were used to fix the osteotomy. Follow up at one year and 10 months showed union of the femur and correction of the shepherd’s crook deformity. He was able to ambulate without walking aid and was painfree. The limb length discrepancy (LLD) was also corrected.

**Fig. 2:**
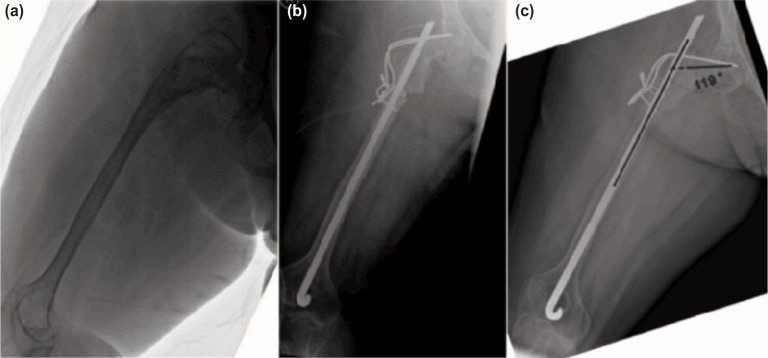
A series of radiographs of the 14-year old girl with osteogenesis imperfecta: (a) The preoperative radiograph shows NSA of less tha 90 degree and external malrotation (b) Immediate post-operative radiograph in Oct, 2013 with subtrochanteric osteotomy to increase NSA and correction of external rotation (c) Radiograph at one year after correction with union, maintenance of corrected NSA and rotation.

The second patient was a 14-year old girl with osteogenesis imperfecta who presented with inability to ambulate without walking frame since sustaining a fracture of the proximal femur six years prior to presentation (Fig. 2). She had never received bisphosphonate. The left lower limb was in an external mal-rotation of 600 and LLD of 7cm. Radiographs showed a mal-united, mal-rotated right femur with NSA of 870 and obvious mal-rotation. We used similar surgical procedure as described below in October, 2013. A Rush rod sized 1F (6.5mm) and two Kirschner wires sized 1.6mm were used to fix the osteotomy. One year after the surgery, the patient ambulated more easily with walking frame. The NSA was maintained at 1100, LLD was improved to 4cm and rotation was corrected. The ability to ambulate has remained good until the most recent phone conversation in May 2017.

We performed the procedure in both patients in the supine position with standard lateral exposure. Transverse osteotomy was carried out at the subtrochanteric level. The proximal segment was reamed retrograde from the osteotomy site using cannulated drill to ensure it started at the lateral most and exited at the greater trochanter. Next, a few Kirschner wires sized 1.6mm were inserted from the lateral femur at subtrochanteric level to the neck. These wires acted as joy-sticks during manipulation of the proximal femur to the required neck-shaft angle (NSA). The distal segment was also reamed using cannulated drill bit to ensure no penetration of the cortex. In the first patient, a Rush rod of selected length and diameter was inserted antegrade from the greater trochanter to the osteotomy site. The Rush rod was then passed through the osteotomy side to distal femur while the NSA was maintained by the Kirschner wires (Fig. 1). In the second patient, the Rush rod was inserted retrograde from the knee joint to greater trochanter while the NSA was maintained by the Kirschner wires. The wires were bent flush on the lateral side of the femur and held with cerclage wires to maintain the NSA and avoid rotation (Fig. 2).

## Discussion

An ideal implant for fixation of osteotomy for this deformity of abnormal bone would be an intramedullary nail with proximal locking screw to the neck to maintain the correction of NSA and distal locking screw to provide stability. Inability to obtain correct diameter and length of intramedullary locking nail at the time of the surgery prompted us to look for an alternative method.

One of the early methods in correcting coxa vara is the Wagner method that involves passing two Kirschner wires through the femoral neck and the external portion of the wires moulded and fixed to the femoral shaft by cerclage wires^2^. In 2003, Fassier improved this technique by combining Wagner’s method with Finidori’s technique which used telescopic rods to fix the subtrochanteric corrective osteotomy^1^. Fassier *et al* in 2008 showed satisfactory outcome with this technique in 18 hips with osteogenesis imperfecta and three hips with fibrous dysplasia ^3^. There was a complication rate of 12% which was largely implant related.

Instead of using telescopic rods, as reported by Fassier *et al*^3^, we modified the technique by using Rush rods as the intramedullary fixation device. Apart from its low cost, it is also a strong solid nail. The Rush rod can be cut according to the desired length as opposed to either intramedullary locking nail or Fassier-Duval nails. Furthermore, in proximal lesions, we were able to control the length of the rod so that it did not breach the distal femur growth plate, as seen in our first patient.

Another difference from the original procedure described by Fassier, was that we did not penetrate the lateral cortex of distal of proximal fragment. Instead, we used cannulated drill bit to create canal in the lateral most part of the shaft. This is possible in an abnormal bone that has big lateral diameter in mal-united fibrous dysplasia bone (first patient) and oval shape osteogenesis imperfecta bone (second patient). If greater valgus correction is required, the penetration through lateral cortex should be chosen. The use of cannulated drill bit is an important step to avoid cortex penetration at unwanted locations especially in deformed and soft osteogenesis imperfecta bone. This method ensures the desired direction of canal to maintain the NSA and avoids the piriformis fossa which might cause injury to important retinacular vessel to the femoral head in immature patients^4^. This modified method provides intramedullary load sharing fixation to the osteotomised bone for long term support. The Kirschner wires that are placed through the neck with the distal half bent and held to the side of the shaft by cerclage wire maintain the NSA as well as prevent rotation. Rotational instability is the limitation faced by the FassierDuval telescopic rod^5^. In order to achieve an acceptable NSA and rotational alignment, the position of cerclage wires must be distal to the osteotomy site. This method provides relative stability to facilitate union. When the nail is properly positioned with a snug fit in the proximal fragment, as seen in the second patient, two Kirschner wires are adequate to maintain NSA and rotation. In case of fibrous dysplasia, the implant may be removed once the lesion has completely healed. In the case of osteogenesis imperfecta, we propose not to remove the implants unless complications arise.

We would also like to highlight that in the second patient with osteogenesis imperfecta, the Rush rod was inserted in a retrograde manner through the knee joint. A retrograde entrance from the knee allows protection of the whole femur from the intercondylar notch where the hook of the Rush rod sits firmly, through the shaft till proximal penetration in the piriformis fossa. We think that it is worth taking the risk of causing joint stiffness with the benefit of reducing the risk of recurrent fracture in osteogenesis imperfecta patients. This patient did not develop any limitation of knee motion. However, the patient should still be warned regarding the possibility of joint stiffness and long term complications.

Based on the experiences gained from these two cases, we are of the opinion that our modified method can be an alternative when an appropriately sized interlocking nail is not available for correction of coxa vara or shepherd’s crook deformity in abnormal bone.
